# Latent Factors Limiting the Performance of sEMG-Interfaces

**DOI:** 10.3390/s18041122

**Published:** 2018-04-06

**Authors:** Sergey Lobov, Nadia Krilova, Innokentiy Kastalskiy, Victor Kazantsev, Valeri A. Makarov

**Affiliations:** 1 Lobachevsky State University of Nizhny Novgorod, Gagarin Ave. 23, 603950 Nizhny Novgorod, Russia; k-nadezhda-k@yandex.ru (N.K.); kastalskiy@neuro.nnov.ru (I.K.); kazantsev@neuro.nnov.ru (V.K.); 2 Department of Applied Mathematics, Instituto de Matemática Interdisciplinar, Universidad Complutense de Madrid, 28040 Madrid, Spain

**Keywords:** electromyography, human–computer interface, motor control, pattern classification, artificial neural networks

## Abstract

Recent advances in recording and real-time analysis of surface electromyographic signals (sEMG) have fostered the use of sEMG human–machine interfaces for controlling personal computers, prostheses of upper limbs, and exoskeletons among others. Despite a relatively high mean performance, sEMG-interfaces still exhibit strong variance in the fidelity of gesture recognition among different users. Here, we systematically study the latent factors determining the performance of sEMG-interfaces in synthetic tests and in an arcade game. We show that the degree of muscle cooperation and the amount of the body fatty tissue are the decisive factors in synthetic tests. Our data suggest that these factors can only be adjusted by long-term training, which promotes fine-tuning of low-level neural circuits driving the muscles. Short-term training has no effect on synthetic tests, but significantly increases the game scoring. This implies that it works at a higher decision-making level, not relevant for synthetic gestures. We propose a procedure that enables quantification of the gestures’ fidelity in a dynamic gaming environment. For each individual subject, the approach allows identifying “problematic” gestures that decrease gaming performance. This information can be used for optimizing the training strategy and for adapting the signal processing algorithms to individual users, which could be a way for a qualitative leap in the development of future sEMG-interfaces.

## 1. Introduction

Multichannel recordings of the surface electromyographic signals (sEMG) allow inferring on the activity of different groups of muscles involved in certain movements [1,2,3,4,5]. Then, each specific movement can be associated with the so-called sEMG-pattern reflecting the degree of contraction of a set of muscles. This, in turn, enables the identification of movements by classification of the sEMG-patterns and, finally, building a human–machine interface based on sEMG recordings [6,7,8,9].

Recent advances in hardware and software for sEMG recording and real-time data analysis fostered the use of sEMG human–machine interfaces for controlling a variety of devices such as, e.g., personal computers [8,10], prostheses of upper limbs [11,12], and exoskeletons [6,7,13,14] among others. Despite the device diversity, the performance of different mathematical strategies involved in the pattern recognition and classification differs only slightly among each other [11,15]. Overall, the performance of sEMG-interfaces has not yet reached the level acceptable for their massive commercial use.

Most methods of extraction of the representative features from sEMG signals are based either on amplitude characteristics and autoregressive models or on the time-frequency analysis and spatiotemporal features [9,16,17]. The pattern classification is usually achieved by linear discriminant analysis (LDA), support vector machines, Bayesian statistics, and artificial neural networks (ANN) [16,18,19,20,21,22,23,24]. One of the most important measures of the efficiency of sEMG-interfaces is the accuracy of motion recognition, which is mostly applicable in synthetic tests. The comparison of different classifiers based on LDA [25,26,27], linear regression models [28], and ANN [9,12,29] has shown that the mean recognition accuracy of rather simple body movements can be high enough. It depends on the number of gestures to be recognized, and may lie in the range (0.93, 0.96) [20,26,30]. In average, diverse approaches may differ by a few percent among each other. However, at the same time, the recognition accuracy and the interface performance may vary significantly (up to 70%) among different users. The latter strongly limits deployment of sEMG-interfaces in society.

The main difficulty in achieving high performance with different persons resides in a wide set of individual characteristics of different subjects, which requires a tedious fine-tuning of interfaces. Moreover, even for the same user, some characteristics may change in time. For instance, the interface performance can degrade significantly due to displacement or shift of recording electrodes, perspiration of skin, fatigue, muscles “crosstalk”, muscle fitness, etc. [10,31]. Thus, a long-term remaining open question is: What are the main factors determining the interface performance? Or more explicitly: Is it the chosen algorithm or the user anatomy, or his/her motor-control ability? An experimentally supported answer to this question may reroute the research efforts directed to solving latent problems of sEMG-interfaces, which could lead to a qualitative leap in their design.

To approach this problem here, we employ two complementary experimental strategies. We investigate the interface performance in synthetic tests (i.e., under single individual gestures) and in a gaming environment. In the former case, we achieve controllable and repeatable conditions, while in the latter, we examine the user experience in “real-life” scenarios. We then systematically study the latent factors influencing the interface performance. In particular, we quantify (i) the degree of muscle cooperation, i.e., the coordinated contribution of synergist and antagonist muscles in a hand movement and (ii) the user’s constitution, i.e., the content of the body fatty tissue. We show that these factors can significantly limit the performance of an sEMG-interface in synthetic tests and provide insight on the social groups of subjects influenced by each factor. We also study how short-term and long-term training can affect the use of interfaces. Surprisingly, the short-term training plays no role in synthetic tests, but significantly increases the gaming performance. We then provide a method for quantification of the gesture recognition fidelity in a dynamic environment. Note that in this case, most of the indexes commonly used in the literature are not applicable. We then discuss the differences between the effects of short-term and long-term training, and how this information can be used for optimizing the training strategy and adapting the signal processing algorithms to the needs of individual users.

## 2. Materials and Methods

### 2.1. Subjects and Short-Term Training

For experimental purpose, we recruited 37 healthy volunteers of either sex (24 women and 13 men) from 18 to 41 years old, and of different fitness and training (12 trained and 25 not trained subjects). In the context of this work by “trained”, we mean persons who regularly practice sport or other activities related to manual small motility (playing guitar, embroidery, etc.). The study complied with the Helsinki declaration, adopted in June 1964 (Helsinki, Finland) and revised in October 2000 (Edinburg, Scotland). The Ethics Committee of the Lobachevsky State University of Nizhny Novgorod approved the experimental procedure (protocol No. 6 from 06.07.2017). All participants gave their written consent. All subjects had no previous experience in dealing with sEMG-interfaces. Fourteen out of 37 subjects (8 women and 6 men) also participated in a ten-day training sessions that consisted in practicing individual synthetic hand gestures and playing a testing arcade game.

### 2.2. sEMG-Interface, “Pacman” Game, and Synthetic Tests

**Figure 1:**
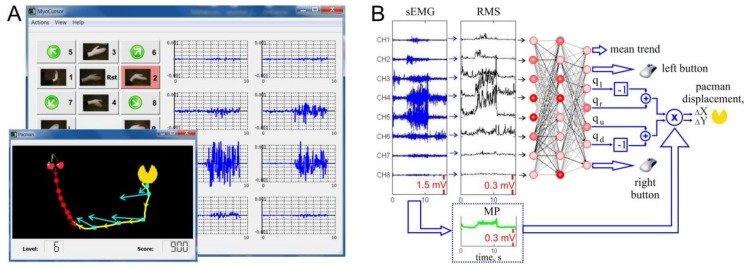
Software design of the sEMG-interface. (**A**) Main window of MyoCursor software (on background) and game window (foreground). A user controls by hand gestures “pacman” (yellow icon) and aims at reaching “cherries” (shown in red), which also can move. In the main window, the current gesture “go left” (G_2_) is shadowed by red. Blue traces show sEMG patterns recorded from eight electrodes of the Myo bracelet. (**B**) Sketch of calculation of the pacman movement by an artificial neural network. Raw sEMG (blue traces) are used to evaluate RMS signals (black traces, Equation (1)). At the same time the mean power (MP) is calculated (green trace, Equation (3)). The artificial neural network classifies RMS patterns and yields an output for controlling the movement direction ($q_{l},q_{r},q_{u},q_{d})$. The “left–right” and “up–down” differences are multiplied by the MP to get proportional control ((ΔX,ΔY) in Equation (5)).

For experimental assessment of an sEMG-interface, we have developed a hardware–software complex called MyoCursor. The system consists of a MYO Thalmic bracelet worn on a user’s forearm, and a PC with a Bluetooth receiver running specially designed software (Figure 1A).

The bracelet is equipped with eight sensors equally spaced around the forearm that simultaneously acquire myographic signals. The signals are sent through a Bluetooth interface to a PC. We used the MYO software development kit to access raw eight-channel data, while the built-in software of the bracelet was disabled. Acquired signals are processed by MyoCursor software v1.12 in real-time. The software recognizes hand gestures and estimates the muscle effort that finally are used for controlling a game module.

#### 2.2.1. Gaming Environment

The game module replicates the well-known arcade game “pacman” (Figure 1A, inset). The user’s objective is to control by hand gestures displacements of pacman on the screen and to catch the “cherry” as quickly as possible.

To control pacman, we selected the following seven hand gestures as basic motor patterns: G_0_, hand at rest (Rst in Figure 1A), was used for relaxation and elimination of а constant trend (see below); G_1_ and G_2_, wrist flexion and extension, imitated movements to the left and to the right, respectively; and G_3_ and G_4_, radial and ulnar deviations, simulated up and down movements, respectively. Besides, we included four additional gestures (5–8 in Figure 1A) that are combinations of pairs of G_1_–G_4_. For example, simultaneous wrist flexion, G_1_, and radial deviation, G_3_, served for diagonal left–up movement. We also used G_9_, hand clenched in a fist (L in Figure 1A) for simulation of the mouse-left click; and G_10_, extended palm (fingers either together or separately, R in Figure 1A) for imitation of the mouse-right click.

#### 2.2.2. Synthetic Tests 

The gaming environment reproduces real-life scenarios of the use of an sEMG-interface. However, due to its dynamic nature, it makes difficult quantification of errors and tuning of the interface. Therefore, in our experiments, we also performed synthetic tests. The subjects were asked to perform sequentially individual static gestures (go left, pause, go right, pause, etc.). The collected data were processed offline. Note that synthetic data also were used for a supervised training of ANN.

### 2.3. Real-Time Processing of sEMG

To process in real-time sEMG signals, we employed two different approaches: (1) ANN-based and (2) linear discriminant analysis (LDA).

#### 2.3.1. ANN Approach 

The data flow $x\left(t\right)\in {\mathbb{R}}^{8}$ was divided into 200 ms overlapping time windows at a 100 ms step (t=0,1,2,… is the discrete time with the sampling rate of 1 kHz). Then, the root mean square (RMS) of the sEMG activity over each time window was evaluated (Figure 1B) [32]:

(1) $$V\left(t\right)=\sqrt{\frac{1}{N}\overset{N-1}{\underset{n=0}{\sum }}x{\left(t-n\right)}^{2}},\;\;\;\;$$

where N=200 is the number of samples in a time window, and t=Mk (k=2,3,4,…) with M=100 being the time shift between consecutive windows. The RMS data, as a composite feature of the current hand gesture, were fed into an ANN with one hidden layer containing eight neurons (Figure 1B).

The network neurons apply weighted sum over their inputs $z\in {\mathbb{R}}^{8}$, and use sigmoidal activation function to generate output y:

(2) $$y=F\left[\left(w,z\right)\right],\;\;\;\;\;\;\;\;\;\;\;F\left(u\right)=\frac{1}{1+e^{-u}},$$

where $w\in {\mathbb{R}}^{8}$ is the vector of the synaptic weights and (⋅,⋅) stands for the inner product. In the output layer, four neurons provide left, right, up, and down values $q={\left(q_{l},q_{r},q_{u},q_{d}\right)}^{T}$ for a given gesture. The learning, i.e., the adjustment of the neuronal weights $\left\{w_{i}\right\}$, is achieved by the standard backpropagation algorithm [33].

Each basic gesture (G_1_–G_4_) corresponds to a single target class. Thus, each output neuron (Figure 1B) should yield 1 for its own class and 0 for the others. To accommodate the compound gestures (e.g., “left–up”, G_6_) during learning, we used the target value $1/\sqrt{2}$ for the corresponding two output neurons. Such a choice allows generating a compound vector output with unitary length for the basic and compound gestures, i.e., ${\left|\left|q\right|\right|}_{2}=1$. Once the learning is deemed finished, the online controlling of pacman can be enabled. To move pacman along the *x*-axis (*y*-axis) we used the value proportional to the difference of the output neurons (Figure 1B) responsible for gestures “left” and “right” (“up” and “down”), i.e., $\left(q_{l}-q_{r}\right)$ and ($q_{u}-q_{d}$).

#### 2.3.2. LDA Approach 

sEMG data can be considered as points in a multidimensional space ${\mathbb{R}}^{N}$. The aim of LDA algorithm is to use a set of hyperplanes (of dimension ${\mathbb{R}}^{N-1}$) to separate the data into different classes. A separating hyperplane is obtained by seeking for the projection maximizing the distance among the means of the classes and minimizing the interclass variance. This technique demands a very low computational power that makes it suitable for online sEMG classification. Earlier studies have shown that the LDA-classifier is quite simple and handy, and in general, provides good results [34]. Similar to the ANN approach, here RMS data were fed to the input of the LDA-classifier implemented in Matlab (function “classify”). First, the routine was configured and then run on the same data used in the ANN approach.

### 2.4. Proportional Control

To introduce proportional control (i.e., depending on the gesture “strength”) we employed an approach similar to that described in References [35,36]. We estimated the muscle effort by evaluating the mean power (MP) over all sEMG sensors [32]:

(3) $$P\left(t\right)=\frac{1}{NK}\sum_{n=0}^{N-1}{\left|\left|x\left(t-n\right)\;\right|\right|}_{1},$$

where K is the number of sEMG channels (in our case K = 8). Then, the pacman speed could be set proportional to the mean power (Figure 1B).

Due to some intrinsic jitter in the muscle tone, we usually observed a slow involuntary drift of pacman on the screen. To eliminate this artifact, the trend defined by the relaxed hand state (Figure 1A, Rst) was subtracted from the pacman controlling signals. Thus, we define the pacman’s velocity by

(4) $$v\left(t\right)=H\left(p\left(t\right)-p_{th}\right),$$

where $p\left(t\right)=P\left(t\right)/P_{max}$ is the relative mean power, $p_{th}$ is the drift threshold, and H(u)=max(0,u) is the rectifier function. Finally, the pacman’s displacement *Δ* along the *x*- and *y*-axes on the screen is given by

(5) $$\Delta \left(t\right)=20v\left(t\right)\left(q_{r}\left(t\right)-q_{l}\left(t\right),q_{u}\left(t\right)-q_{d}\left(t\right)\right).$$

### 2.5. Performance of sEMG Interface

For estimating the performance of the sEMG-interface in synthetic tests, we employed two measures. The first was the so-called *F*-measure [37], which is based on the precision and recall values obtained from the classification results [32]:

(6) $$P=\frac{TP}{TP+FP},\;\;R=\frac{TP}{TP+FN}\;,$$

where *TP* is the number of true positives, i.e., correctly recognized gestures; *FP* is the number of false positives, i.e., a classifier recognizes other gesture as its own; and *FN* is the number of false negatives, i.e., a classifier does not recognize its own gesture. Then, the *F*-measure is given by

(7) $$F=\frac{2PR}{P+R}\;.$$

This measure is convenient for quantifying the interface performance in synthetic tests, since it can be calculated both for each gesture separately, and for all of them together. Note that in the latter case, a classifier is tested on “known” gestures, hence FN = 0 and thus R = 0.

We used the *F*-measure for comparing the performance of the ANN and LDA classifiers in the task of classification of four main gestures G_1_–G_4_. However, the compound gestures (G_5_–G_8_) require different approach. Indeed, in this case, the output of the ANN is not binary, and thus the assignment of *TP*, *FP,* and *FN* is not straightforward. We then used the mean squared error of the difference between the network output q and target classification u. The error was evaluated over N trials and *M* neurons in the last layer (in our case *M* = 7):

(8) $$E_{MS}=\;\frac{1}{NM}\overset{N}{\underset{n=1}{\sum }}|\left|q_{n}-u_{n}\right|{|}_{2}^{2}.$$

The mean squared error (8) was calculated for the network training and testing sets. It served as a criterion to stop the learning procedure, as soon as the error started increasing on test samples. On average, the learning process required about 5000 training epochs and took less than 1 min on a standard Intel Core i5 PC.

Our experimental data show that $E_{MS}$ varies strongly from one person to another in the range (0.009, 0.054). The empirical distribution of $E_{MS}$ deviates significantly from a normal distribution (a Lilliefors test rejected the null-hypothesis of normality, p = 0.04; median $E_{m}=\;0.022$, quartiles $Q_{1}=0.017$ and $Q_{3}=0.027$). Thus, to use parametric statistics, we normalized the distribution by applying logarithmic transformation and introduced the performance index:

(9) $$Re=\ln\left(\frac{E_{MS}}{E_{m}}\right).$$

The distribution of Re was close enough to a Gaussian distribution (a Lilliefors test accepted the null-hypothesis of normality, p > 0.5).

### 2.6. Assessment of Factors Influencing Performance of sEMG-Interface in Synthetic Tests

#### 2.6.1. Body Fat (BF) Index

To test the correlation between the classification error and anatomic features of the users, for each subject we estimated the amount of fatty tissue in the body by the fat monitor OMRON BF306. Personal anthropometric characteristics, i.e., weight, height, age, and sex were introduced into the analyzer. The device measures the impedance from hand to hand and calculates the body fat (BF) in percentage based on the collected data. In our study, the subjects had the BF index in the range (4, 44)%.

#### 2.6.2. Synergist-Antagonist Coefficient (SAC)

Each body movement involves contraction and extension of various muscles. These concurrent processes must be perfectly synchronized and tuned to perform movements optimal in terms of the energy consumption and precision. To verify the influence of the “muscle functional efficiency” of a subject on the sEMG-interface performance, we developed a novel measure: the coefficient of activation of synergist-antagonist muscles or SAC. It will be thoroughly discussed in Section 3.2.

### 2.7. Quantification of Interface Performance in Gaming Environment

To study the performance of the sEMG-interface in real-life scenarios, we conducted experiments with the pacman game. While playing, the subjects freely move their hands, thus we get no clear reference on the gesture performed in each time instant. Therefore, the above-described indexes are not applicable directly. We then developed a measure based on an analysis of trajectory of pacman controlled by users while persecuting cherries (Figure 1A, inset).

At each game trial, the trajectories of pacman and of cherries (Figure 1A, inset, shown in yellow and red) where acquired together with the gestures provided by the ANN (variable q(t)). These data were downsampled in such a way that each trajectory, i.e., the position of pacman at given time instant $\left\{\rho_{p}\left(t_{i}\right)\right\}$, had no more than 60–100 points (about 50 ms between consecutive points). Then, we estimated the velocity vector, i.e., the direction of motion of pacman by finite difference:

(10) $$\omega_{p}\left(t_{i}\right)=\frac{\rho_{p}\left(t_{i+1}\right)-\rho_{p}\left(t_{i}\right)}{t_{i+1}-t_{i}}\;\;.\;$$

The same procedure was applied to the trajectory of cherries yielding the location $\rho_{c}\left(t_{i}\right)$ and the velocity $\omega_{c}\left(t_{i}\right)$. Using these data, we can evaluate the quality of the decision made by the user.

To get a reference, we have to calculate an optimal direction of motion, i.e., the direction of pacman providing the best intercepting strategy. There are several approaches to this problem from simple strategies to cognitive models (see, e.g., [38,39,40]). We, however, applied the simplest algorithm sufficient for the fastest target interception. First, we solve the following equation for the interception time $t^{*}>0$:

(11) $$\left|\left|\omega_{p}\left(t_{i}\right)\right|{|}_{2}t^{*}=\right|\left|\rho_{c}\left(t_{i}\right)-\rho_{p}\left(t_{i}\right)+\;\omega_{c}\left(t_{i}\right)t^{*}\right|{|}_{2}\;.\;$$

Then, we calculate the unit vector:

(12) $$n\left(t_{i}\right)=\frac{\rho_{c}\left(t_{i}\right)-\rho_{p}\left(t_{i}\right)+\;\omega_{c}\left(t_{i}\right)t^{*}}{|\left|\rho_{c}\left(t_{i}\right)-\rho_{p}\left(t_{i}\right)+\;\omega_{c}\left(t_{i}\right)t^{*}\right|{|}_{2}},\;$$

defining the best direction the user can take at time instant $t_{i}$. Note that in certain cases (e.g., small pacman’s velocity) Equation (11) can have no solution. Then, as the best direction, we take

(13) $$n\left(t_{i}\right)=\frac{\rho_{c}\left(t_{i}\right)-\rho_{p}\left(t_{i}\right)+\;\omega_{c}\left(t_{i}\right)\left(t_{i+1}-t_{i}\right)}{|\left|\rho_{c}\left(t_{i}\right)-\rho_{p}\left(t_{i}\right)+\;\omega_{c}\left(t_{i}\right)\left(t_{i+1}-t_{i}\right)\right|{|}_{2}}.\;$$

Given the best direction (12) (or (13)) and the real direction of pacman movement, we can evaluate the angular deviation $\alpha \left(t_{i}\right)=∠\left(\omega_{p}\left(t_{i}\right),n\left(t_{i}\right)\right)$, which quantifies the error of the user action at time instance $t_{i}$. Further, we apply the circular statistical analysis to describe the distribution of $\left\{\alpha \left(t_{i}\right)\right\}$ at a single game trial or by averaging at a single game level, or overall for the game.

To evaluate the fidelity of gestures identified by the ANN, we use the ANN output vector (Figure 1B):

(14) $$\delta \left(t_{i}\right)=\left(q_{r}\left(t_{i}\right)-q_{l}\left(t_{i}\right),q_{u}\left(t_{i}\right)-q_{d}\left(t_{i}\right)\right).$$

Then, the gesture at $t_{i}$ is considered optimal if the following inequality holds:

(15) $$\frac{\left(\delta \left(t_{i}\right),n\left(t_{i}\right)\right)}{|\left|\delta \left(t_{i}\right)\right|{|}_{2}}>d_{th},$$

where $d_{th}=0.6$ is the threshold of angular deviation from the best decision (angles within 0.92 rad cone). Otherwise, the gesture is classified as incorrect. Next, the optimal and incorrect gestures are divided into groups with prevalence of “left”, “right”, “up”, and “down” components. For example, the set of “optimal left” gestures is defined by

(16) $$\Omega_{left}^{opt}=\left\{i:\;\;\left(\delta \left(t_{i}\right),n\left(t_{i}\right)\right)>d_{th}|\left|\delta \left(t_{i}\right)\right|{|}_{2},\delta_{1}\left(t_{i}\right)<-0.2\sigma \right\},$$

where σ is the standard deviation of $\delta_{1}$. The other sets $\Omega_{right}^{opt}$, $\Omega_{left}^{inc}$, $\Omega_{right}^{inc}$, $\Omega_{up}^{opt}$, $\Omega_{up}^{inc}$, $\Omega_{down}^{opt},\;$and $\Omega_{down}^{inc}$ are defined similarly to (16). Finally, we estimate the gesture rates as the relative cardinality of the sets. For example:

(17) $$R_{left}^{opt}=\frac{\left|\Omega_{left}^{opt}\right|}{L},$$

where L is the total number of gestures.

## 3. Results

### 3.1. General Performance of sEMG-Interface and Short-Term Training

**Figure 2:**
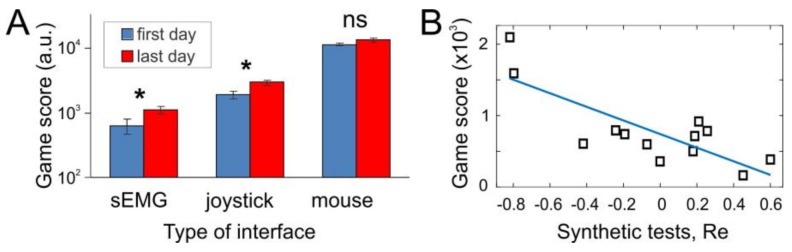
General performance of sEMG-interface. (**A**) Comparative analysis of different types of interfaces in gaming environment in the first day of experiments and after a ten-day training. The training practically doubled the score obtained by the sEMG-interface, significantly increased it for joystick, and no significant changes were observed for computer mouse. (**B**) Correlation between two experimental paradigms testing sEMG-interface: the game score vs the performance index *Re* in synthetic tests for individual subjects (squares). Straight line represents linear regression (p=0.001).

To estimate the general performance of the sEMG-interface, we conducted experiments with the pacman game (see Methods). In the same test to drive pacman, the subjects also used more common interfaces: a joystick, and a computer mouse. To get insight on the effect of short-term learning, testing sessions were repeated during ten days. Figure 2A summarizes the results of the game score obtained by the subjects.

As was expected, we obtained quite diverse game scores for different types of interfaces. Note that the hand movements, and hence sEMG patterns, are quite similar for sEMG-interface and joystick, and differ significantly from the mouse control. Computer mouse was the handiest for playing the game. However, to our surprise, results shown with joystick (i.e., under direct control of pacman) were much closer to the sEMG-interface than to mouse (note logarithmic scale in Figure 2A). This observation suggests that the human abilities for handling different types of interfaces depend strongly on long-term training. Indeed, the subjects participated in the experiment were wont to use mouse in their daily life, and much less accustomed to joystick. Thus, we expect that quite low game performance reached with the sEMG-interface may be improved significantly by long-term training. Our data support this hypothesis. After а short-term training lasting 10 days (14 subjects) the game score obtained with the sEMG-interface practically doubled its initial value. The performance with joystick also increased significantly, whereas no changes were observed with mouse (Figure 2A). Thus, the incremental improvement is inversely proportional to the previous experience obtained with the interface before the experiments.

Besides testing the sEMG-interface in the gaming environment, we also evaluated its performance in synthetic tests while subjects were performing separate individual gestures (G_1_–G_8_). In such a case, we can evaluate the performance index *Re*, which quantifies the error at the neural network output. Then, we study the correlation between two types of experimental approaches. Each individual subject first performed synthetic gestures, and we evaluated *Re* and then the same subject played the pacman game, and we recorded the obtained score. Figure 2B shows the obtained data and results of linear regression:

(18) Score=a×Re+b,  a=−952±219,  b=741±93,

which confirmed the strong correlation between the selected measures (p = 0.001). Thus, we can conclude that two experimental approaches (synthetic and gaming tests) provide complementary data and can be used in parallel.

### 3.2. Synergist–Antagonist Coefficient (SAC)

**Figure 3:**
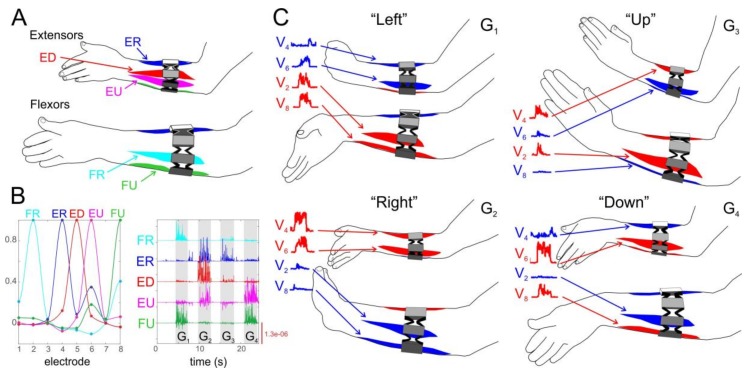
Synergist–antagonist coefficient describing the muscle functional efficiency. (**A**) Sketch of five main muscles (in two mirrored projections) involved in synthetic gestures G_1_–G_4_: *extensor carpi radialis longus* (ER), *extensor digitorum* (ED), *extensor carpi ulnaris* (EU), *flexor carpi radialis* (FR), and *flexor carpi ulnaris* (FU). (**B**) Independent component analysis of an sEMG recording. The loadings of five components (left) and the corresponding activations (right), while a subject performs four main gestures G_1_–G_4_. (**C**) Traces of activations of two synergist and two antagonist muscles (in red and blue, respectively) for each of the gestures G_1_–G_4_.

The MYO bracelet records sEMG signals from a forearm, which has a number of muscles that participate in performing different gestures (Figure 3A). We then identified those of them that significantly contribute to the synthetic gestures G_1_–G_4_. To this end, the sEMG recordings have been processed by independent component analysis (ICA), which has been previously shown to be effective for the analysis of multielectrode recordings of local field potentials (LFP) [41,42,43]. There is an important similarity between LFP and sEMG. Indeed, in both cases, electrical signals are generated by various sources (neuronal and muscle membranes for LFP and sEMG, respectively) and are mixed on external electrodes (extracellular and surface for LFP and sEMG, respectively). The data model in the case of sEMG (similar to LFP) can be written in the following form:

(19) $$V\left(t\right)=\overset{m}{\underset{k=1}{\sum }}W_{k}s_{k}\left(t\right),$$

where $V\left(t\right)\in {\mathbb{R}}^{8}$ are the RMS of the sEMG signals, $\left\{W_{k}\right\}$ is the set of loadings (weight vectors), and $\left\{s_{k}\left(t\right)\right\}$ are the time activations. Thus, the recorded signals are represented as a linear combination of contributions from m sources (muscles). The ICA estimates both the loadings and time activations from the original data.

Our studies have shown that sEMG signals are mainly contributed by five sources, i.e., m=5 (Figure 3B). Moreover, these sources are well localized in space (loadings $W_{k}$ strongly peaked at certain electrodes) and coincide with anatomical location of five muscles (Figure 3A,B): (1) *flexor carpi radialis* (FR), (2) *flexor carpi ulnaris* (FU), (3) *extensor carpi radialis longus* (ER), (4) *extensor digitorum* (ED), and (5) *extensor carpi ulnaris* (EU). Other muscles (e.g., *palmaris longus*) may also contribute to gestures G_1_–G_4_ and, consequently, to sEMGs, but their signals are weak enough and can be neglected while dealing with the SAC.

Thus, given that the MYO bracelet has been placed correctly on a subject’s forearm, we can accept that electrodes 2, 4, 5, 6, and 8 capture exclusively the activity of the corresponding main muscles. Figure 3B (right) shows the activation of the independent components (main muscles) when a subject performs gestures G_1_–G_4_. The activity exhibits clear patterns for each of the four gestures.

For the sake of simplicity and taking into account the symmetry of activations, we selected four muscles out of five: FR, ER, EU, FU (Figure 3A). Depending on the hand gesture, these muscles can act either as synergists or antagonists. The quantification of the contribution of synergist and antagonist muscles has been earlier used by Kurenkov and colleagues [44] for optimizing injection of toxins in clinical practices. We here take the activities in electrodes 2, 4, 6, and 8 as the reference of the contraction of the corresponding muscles, and calculate the mean RMS values over several samples for four basic gestures G_1_–G_4_:

(20) $$\bar{V}=\frac{1}{M}\overset{M}{\underset{t=1}{\sum }}V\left(t\right).$$

Within our approach, each gesture has two synergist muscles and two antagonist ones. We thus introduce the synergist S and antagonist A indexes by using the corresponding elements of the vector $\bar{V}\in {\mathbb{R}}^{8}$ (Figure 3C):

- G_1_ (“left”): $S_{1}={\bar{V}}_{2}+{\bar{V}}_{8}$, $A_{1}=\;{\bar{V}}_{4}+{\bar{V}}_{6}$;
- G_2_ (“right”): $S_{2}={\bar{V}}_{4}+{\bar{V}}_{6}$, $A_{2}=\;{\bar{V}}_{2}+{\bar{V}}_{8}$;
- G_3_ (“up”): $S_{3}={\bar{V}}_{2}+{\bar{V}}_{4}$, $A_{3}=\;{\bar{V}}_{6}+{\bar{V}}_{8}$;
- G_4_ (“down”): $S_{4}={\bar{V}}_{6}+{\bar{V}}_{8}$, $A_{4}=\;{\bar{V}}_{2}+{\bar{V}}_{4}$.

Then, the ratio $S_{k}/A_{k}\in \left(0,\infty \right)$ reflects the muscle functional efficiency, while a subject performs gesture k. Finally, the synergist–antagonist coefficient for a subject is given by averaging the ratios over all *M* gestures:

(21) $$\mathrm{SAC}=\ln\left(\frac{1}{M}\overset{M}{\underset{k=1}{\sum }}S_{k}/A_{k}\right).$$

Note that the logarithmic scaling in Equation (21) serves for normalization of the coefficient. Then SAC = 0 means that synergist and antagonist muscles are equally activated by the gesture, whereas SAC > 0 (SAC < 0) indicates prevalence of synergetic (antagonistic) muscles’ contraction. We thus expect that higher SAC values correspond to better coordination of muscles, while performing different gestures and hence should result in a lower error rate of the sEMG-interface.

**Figure 4:**
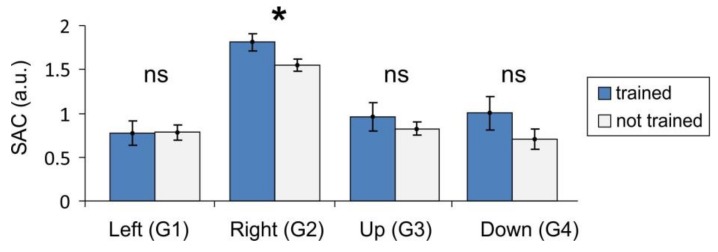
Synergist–antagonist coefficient (SAC) for trained and not trained subjects (mean values and standard errors are shown; star marks statistically significant difference).

Figure 4 shows the SAC obtained in groups of physically trained and not-trained people for each of the main gestures G_1_–G_4_. We remind that by “trained”, we mean persons who regularly practice sport or other activities related to manual small motility. For three gestures out of four (G_2_–G_4_) the mean value of the SAC is higher for trained people, as we expected. For G_1_ (wrist flexion), the means are practically the same. This is because G_1_ is the most natural gesture that does not require strong muscle activation. On the available data, the statistically significant difference appears in the case of G_2_ only (Figure 4, *t*-test, *p* = 0.03). Note that this is the gesture (wrist extension) with the maximal SAC, which requires strong muscle activation and coordination. Non-significant differences observed in other cases may be due to not sufficient statistics.

### 3.3. Similar Means and High Variance of sEMG Performance for Different Classifiers

Above, we mentioned that different types of classifiers usually provide similar performance when applied to sEMG signals. Let us now confirm this observation in an example of the ANN and LDA classifiers.

**Figure 5:**
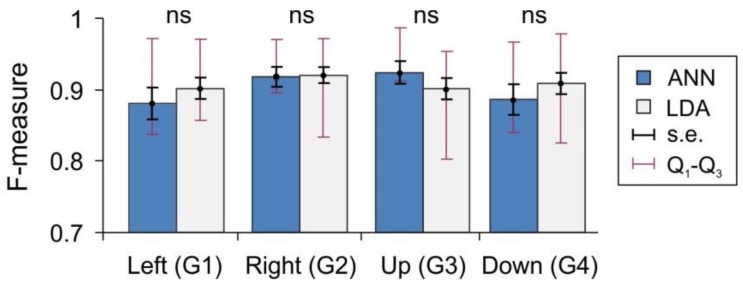
Two classifiers artificial neural network (ANN) and linear discriminant analysis (LDA) show similar recognition performance for gestures G_1_–G_4_ (all differences are not significant, ns).

Figure 5 shows the *F*-measure of the gesture recognition fidelity (see Materials and Methods) for the ANN and LDA classifiers applied over the same data set (subjects performing synthetic gestures G_1_–G_4_). For both classifiers, the mean values lie in the rather narrow interval (0.88, 0.95). However, the dispersion of the measure (interquartile Q_1_–Q_3_ intervals) over different subjects is quite high, in the range (0.8, 0.98). Note that the lower bound *F* = 0.8 corresponds to a strongly uncomfortable situation for a user. The LDA method performs slightly better than the ANN on gestures G_1_ and G_4_, equally well on G_2_, and worse on G_3_. Nevertheless, the statistical analysis shows no significant difference between the classifiers. Thus, the equal means and high dispersion of the performance suggest that, to a great extent, the limiting factors for sEMG-interfaces can be related to the individual properties of different subjects, and to a lesser extent, to the type of classifier. Then, a perfect classifier should take into account the individual user’s properties.

### 3.4. Latent Factors Influencing sEMG Performance

It is reasonable to assume that long-term training of hand muscles in daily life can lead to a more efficient motor control. Eventually, it will be reflected in more coordinated sEMG-patterns and, consequently, will lead to a better performance of the sEMG-interface. Let us now crosscheck this hypothesis.

**Figure 6:**
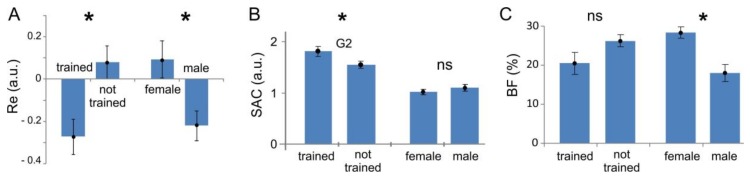
Assessment of latent factors influencing the performance of the sEMG-interface (mean values and standard errors are shown; stars mark statistically significant changes, *p* < 0.05; ns stays for not significant). (**A**) Performance index (relative error), Re; (**B**) Synergist–antagonist coefficient, SAC; (**C**) Body fat index, BF.

Figure 6 shows the statistic for the performance index (panel A), synergist–antagonist coefficient (panel B), and body fat index (panel C) for different groups of subjects. We observe a statistically significant difference in the performance index between physically trained and not-trained people, and also between men and women (Figure 6A, *t*-test, p = 0.002 and p = 0.01, respectively). As we have seen above, the SAC for the most demanding gesture G_2_ exhibits statistically significant difference between trained and not trained subjects (Figure 6B, *t*-test, p = 0.03). However, there is no statistically significant difference between men and women (*t*-test, p = 0.5). Oppositely, the body fat measure differs significantly between men and women (Figure 6C, *t*-test, p = 0.0004) and non-significantly between trained and not trained subjects. Thus, they are two complementary indices. The SAC is not sensitive to the body fat, but explains better muscle coordination in trained people.

Let us now go into detail of the observed gross differences in the performance index between different user groups (Figure 6A). The difference should be associated with some latent factors, individual for each subject. Here, we test the level of coordination of muscles (represented by the SAC) and the percentage of fatty tissue (represented by the BF index). We then correlated the coefficients evaluated individually for each subject with the achieved performance.

**Figure 7:**
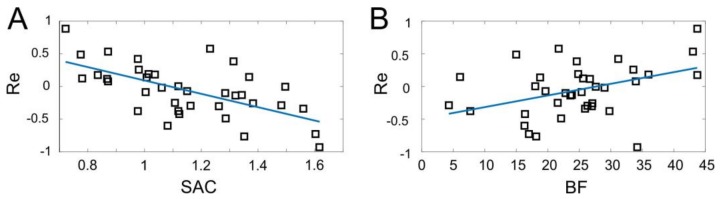
Performance of the sEMG-interface as a function of latent factors: (**A**) The synergist–antagonist coefficient (SAC) and (**B**) body fat index (BF) (squares). In both cases, there is a significant correlation. Blue lines represent linear regressions (p = 0.001 and p = 0.01 for (**A**) and (**B**), respectively).

Figure 7 shows the results of linear regressions. For the dependence of Re on SAC we obtained the following straight line (Figure 7A, p = 0.001):

(22) Re=a×SAC+b,  a=−1.02±0.22,  b=1.11±0.26.

Thus, the performance of the sEMG-interface depends significantly on the muscle control efficiency. The error of gesture identification decreases with an increase of SAC.

The next question we addressed was the observed difference in the performance between men and women (Figure 6A). Note that it cannot be explained directly by the muscle efficiency (SAC), since it is similar between men and women (Figure 6B). Then, we assume that it may be explained by the variation in the body composition and, especially, by the content of fat tissue, which is significantly higher in women (Figure 6C). Indeed, relating the performance and the BF index we revealed a statistically significant correlation (Figure 7B, p = 0.01). The linear regression of the data provides:

(23) Re=α×BF−β,     α=0.018±0.007,     β=0.5±0.18.

Thus, the error of gesture identification increases with an increase of the body fat, which explains, at least partially, the worse performance of female users (Figure 6A,C).

### 3.5. Short-Term Training in Gaming Environment

Above (Figure 2A), we have observed that practicing the pacman game with the sEMG-interface during ten days led to a significant increase of the game score. We, however, did not find a significant difference both in the performance index *Re* and in the synergist–antagonist coefficient SAC in the synthetic gesture tests before and after the short-term training. Thus, the increase in the gaming performance can be caused by latent factors other than those captured by these indexes. We then hypothesize that the gaming improvement may be implemented at a higher decision-making level, which is not relevant for pure gestures.

**Figure 8:**
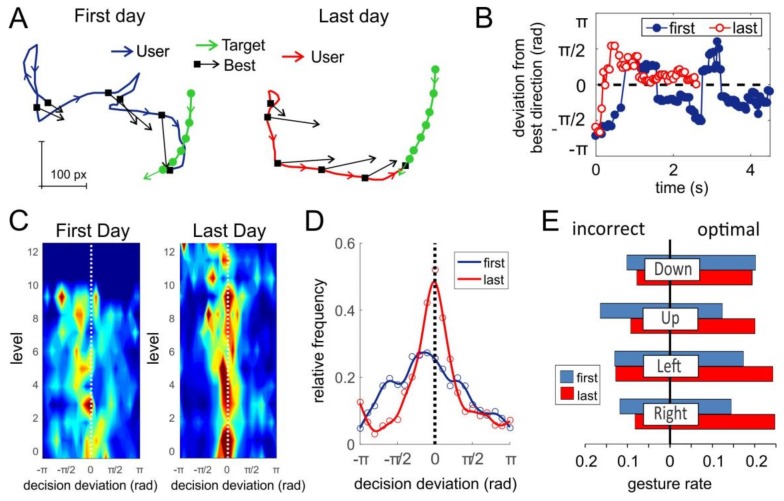
Analysis of the gaming performance of a single subject playing the pacman game at the first day and after ten-days training. (**A**) Representative example of two game trials (in the first day and after training). Green arrowed curves show the movement of the target. Blue and red curves correspond to the trajectory of pacman. Black arrows mark the best game decisions along trajectories. (**B**) Deviation of the user choice from the best gaming decision along the trajectories shown in (**A**). (**C**) Histograms of the decision deviation from best direction along game levels (color from blue to red represents the frequency of the corresponding deviation). (**D**) Relative frequency (probability) of the trajectory deviation from the optimal direction. (**E**) Rates of incorrect and optimal gestures used for controlling pacman before and after training.

To test this hypothesis, we performed a comparative analysis of the decisions made by users while playing the pacman game before and after short-term training. Figure 8A illustrates a representative example of two game trials at the first day (left) and after the training (right). In both cases the target (cherry in inset in Figure 1A) moves along similar trajectories (green curves in Figure 8A). Pacman controlled by the user starts persecution of the target also from similar positions. Therefore, we have similar gaming scenarios. However, in the first day, the pacman’s trajectory is significantly more twisted than in the last day (blue and red curves in Figure 8A, respectively). This suggests that training with the sEMG-interface improves the quality of controlling of pacman, which in turn leads to a higher scoring.

To quantify the controlling quality, we estimated the best gaming decisions (see Materials and Methods) at several points of pacman’s trajectories, i.e., the directions of pacman movement that would lead to the fastest target interception (Figure 8A, black arrows). One can observe that the pacman trajectories deviate from the best decisions. Then, we calculated the angular error (deviation) of the user trajectory from the best direction (Figure 8B). As we expected, the deviation obtained in the first day strongly oscillated staying far away from best directions, which led to scouring and a zigzag-like behavior. After the short-term training, the angular deviation was much closer to zero (best course) and hence the user achieved faster interception (4.5 s in the first day vs 2.4 s in the last one).

We then averaged the results shown in Figure 8A,B over all trajectories at different game levels. Figure 8C illustrates histograms of the decision deviation for different game levels. Indeed, training increases the frequency of optimal decisions (red color around zero deviation). This allowed the user to reach level 12 after the short-term training vs level 9 at the first day. Figure 8D shows the overall relative frequency (estimated probability) of the decisions made by the subject in the first and in the last days. We observe that the short-term training significantly improved the quality of control of pacman by the sEMG-interface. The distribution in the first day is notably wider. Moreover, the peak (most frequent decision) is shifted to negative angles, which means that in the first day, the user had a bias to turn left from the best direction.

The discussed statistics for a single user confirms that short-term training can improve the user experience with the sEMG-interface. However, it does not shed light on the question of the way it happens. To get additional information on the user’s decisions, we separated gestures identified by the ANN controlling the movement of pacman into “optimal” and “incorrect”, according to the angle between the user’s selected and the best directions. Then, we identified the rates of “optimal” and “incorrect” right, left, up, and down gestures. Note that in a gaming environment, the definition of pure gestures cannot be introduced. Instead, we applied a threshold criterion to the ANN output (see Materials and Methods).

Figure 8E shows the rates of optimal and incorrect gestures for the selected subject. We observe that in the first day the user had serious problems with articulating gesture “up”. This gesture has the highest incorrect rate and the lowest optimal rate in the first day. We note that these problems were not caused by bad recognition of gesture “up” by the classifier. Its overall rate (length of the blue bar) is similar to other gestures. The reason probably is an excessive delay of evoking this gesture by the user, and as a consequence, the gesture appears late, and hence is identified as incorrect in the dynamic game.

The articulation of gestures improves after the short-term training. The most notable result was obtained in the problematic gesture “up”. Its incorrect rate decreased while the optimal one increased significantly. Besides, the user improved the rates of optimal gestures “right” and “left”. We note that the experiment was “blind”, i.e., the user was not alerted after the first day about the problem he had with gesture “up”. Nevertheless, in a commercial use of an sEMG-interface, such a knowledge could be useful for a user, and may allow reaching better training results. We also note that in our experiments we observed that different users had problems with different gestures. Thus, the training process should be individual. It also means that efforts to improving the sEMG-interface can be directed to the interface customization for individual users.

**Figure 9:**
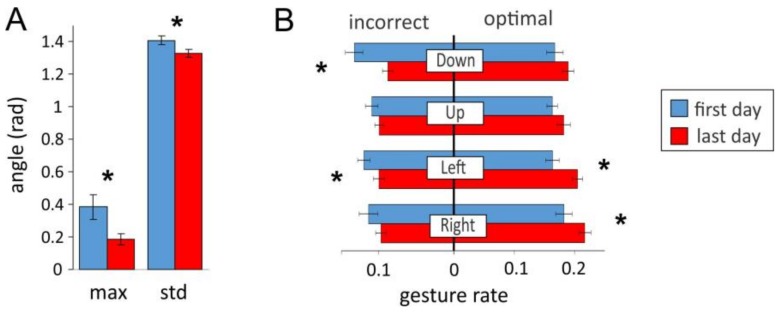
Overall improvement in controlling pacman by the sEMG-interface after short-term training. (**A**) Mean characteristics of the angular deviation from best decisions: location of the maximum (most frequent decision) and the standard deviation of the distributions (see also Figure 8D). (**B**) Rates of incorrect and optimal gestures (stars mark statistically significant changes).

Figure 9 shows the results averaged over fourteen subjects. We found statistically significant decrease of the angular deviation of the most frequent decision from zero and of the standard deviation of the distribution after the training (Figure 9A, see also Figure 8D). The short-term training also led to a better gesture articulation (Figure 9B). The rates of all incorrect gestures decreased (statistically significant for “down” and “left”), and the rates of all optimal gestures increased (statistically significant for “left” and “right”).

## 4. Conclusions and Discussion

In this work, we have systematically studied the latent factors determining the performance of sEMG-interfaces. To this end, we have employed two complementary experimental strategies. On the one hand, we tested the interface performance in a gaming environment, which allowed us to examine the user experience in scenarios close to real ones. The developed “pacman” game also permitted keeping the motivation of subjects during short-term training lasting ten days. On the other hand, to discard the influence of factors extrinsic to the interface features (e.g., gaming strategies) and to work in controllable and repeatable conditions, we also performed synthetic tests. In this case, the subjects were asked to repeat a set of individual gestures (move left, move right, etc.).

The subjects recruited for experiments were wont to use computer mouse in their daily life, and much less accustomed to joystick, and had never used the sEMG-interface. The gaming tests showed that short-term training with the sEMG-interface practically doubled the game score achieved by the users. At the same time, the results obtained with joystick also showed a significant increase, while playing with computer mouse revealed no changes. Moreover, the mean game score achieved with joystick was much closer to the sEMG-interface than to mouse. Thus, the game design was appropriate, since it minimized the influence of gaming skills, i.e., a high-level reasoning, and unveiled features exclusively related to the interfaces. The success in the short-term training obtained with the sEMG-interface allows us foreseeing its high potential, given that appropriate training conditions will be met.

Then, we have analyzed the latent factors determining the sEMG-interface performance in synthetic tests. We thus introduced (a) the performance index *Re*, which quantifies the error at the neural network output; (b) the *F*-measure, which estimates the rate of correctly and incorrectly identified gestures; and (c) the synergist–antagonist coefficient (SAC), which reflects the muscle functional efficiency. Our gross results have confirmed the earlier reported data [20,30,34,45] stating that the performance of sEMG-interfaces can vary significantly from person to person. For example, the interquartile Q_1_–Q_3_ interval of the *F*-measure lies in the range (0.8, 0.9). The lower limit (*F* = 0.8) makes the use of an interface strongly uncomfortable for a user. These figures have been obtained with two different classifiers based on linear discriminant analysis and artificial neural networks. At different gestures, the LDA method performed either slightly better or worse than ANN, but we observed no statistically significant difference between the classifiers. This suggests that a qualitative leap in the sEMG-interface performance may require novel approaches to the user training or ANN post-training procedures. A promising approach in this direction can be based on the novel concept of “high-dimensional brain” [46].

Analyzing different user groups in synthetic tests, we found statistically significant differences between men and women and between physically trained and not-trained subjects. The higher interface performance found for men can be linked to the content of fat tissue in the body. Earlier, this factor has been discussed in several studies (for review see, e.g., [47]). Fat tissue decreases the conductance of bioelectric potentials, and hence, it influences the amplitude of sEMG signals. This in turn reduces the signal-to-noise ratio and, as a consequence, the fidelity of gesture identification. Indeed, in our study we have revealed a statistically significant correlation between the classification error *Re* and the body fat index.

Next, we focused on investigating the impact of unspecific (i.e., not related to the sEMG-interface) long-term training of users. We selected a group of “trained” subjects regularly practicing sports or other activities involving manual small motility (e.g., playing guitar, embroidery). This group consisted of men and women, and had a decreased body fat index. However, the decrease was not significant. Thus, we hypothesized that the difference observed between physically trained and not-trained people cannot simply be reduced to the body fat index, but is also explained by the degree of functional muscle cooperation.

Using independent component analysis, we have shown that sEMG signals are mainly contributed by five sources that coincide in space with anatomical location of five muscles: *flexor carpi radialis*, *flexor carpi ulnaris*, *extensor carpi radialis longus*, *extensor digitorum*, and *extensor carpi ulnaris*. For each individual gesture, we defined synergist and antagonist muscles and evaluated their activation ratio, SAC. We thus expected that higher SAC values should correspond to better coordination of muscles, and hence, should result in a lower error rate of the sEMG-interface. We have checked that the SAC is not sensitive to body fat, and hence can be used to contrast our hypothesis.

We have shown that the success in handling the sEMG-interface indeed depends on the SAC of a subject. For three out of four gestures, the mean value of the SAC was higher for trained people, as expected. The means for the fourth gesture “go left” (G_1_) were the same, because it is the most natural gesture that requires no strong muscle activation. Thus, the difference between physically trained and not-trained subjects besides the body fat index can be explained by long-term training of hand muscles, and related brain circuits involving motoneurons, which lead to a more efficient motor control.

Practicing the pacman game with the sEMG-interface during several days led to an important increase of the game score. This effect was common for all users, and thus, could not be explained by the abovementioned reasons. We then focused on investigating the impact of short-term training. Surprisingly, we did not find significant differences both in the performance index *Re* and in the synergist–antagonist coefficient in synthetic tests before and after the training. Thus, in contrast to the long-term training mostly affecting muscles and low-level neural circuits, the increase in the gaming performance could be caused by latent factors working at a higher decision-making level, which are not relevant for synthetic gestures.

To test this hypothesis, we performed a comparative analysis of trajectories of pacman before and after the short-term training. To do that, we estimated the best gaming decisions, i.e., the direction of pacman movement leading to the fastest target interception. Then, we have shown that the deviation of the user’s trajectory from the best direction in the first day was significantly stronger than after the short-term training. To get additional insight on the quality of the user’s decisions, we classified gestures identified by the neural network into “optimal” and “incorrect” in accordance with the deviation from the best direction.

The most notable result was the finding that different subjects have different “problematic” gestures. After the short-term training, all users improved the rates of optimal gestures and decreased the rates of incorrect ones. On average, the rates of all incorrect gestures decreased (statistically significant for “down” and “left”) and the rates of all optimal gestures increased (statistically significant for “left” and “right”). We note that such a result was obtained in “blind” experiments, i.e., the subjects were not alerted after the first day about the problems they might have. Nevertheless, in a commercial use of sEMG-interfaces, such knowledge could be useful for optimizing the training process by paying strong attention to problematic gestures. Thus, the training process and efforts to improving sEMG-interfaces should be directed to their individual tuning.

Overall, the obtained data suggest that short-term training can improve the interface performance by some plastic changes occurring at the upper cognitive level. To achieve progress at the low level of muscles and motoneurons, long-term training is required. However, such training demands strong motivation from a user. In our experiments, we observed a significant drop in motivation already after ten days. In this respect, it seems promising to study sEMG-interfaces with amputees who do not have the opportunity to use standard interfaces. In this case, the long-term training may provide social rehabilitation and improvement of the life quality through access to online services. Then, the mechanisms of transferring of skills acquired in short-term training to long-term neuromotor synchronization can be revealed.

Finally, to support our conclusions, we recall EEG studies of motor performance. In particular, it was shown that EEG of athletes exhibits changes depending on the kinematic characteristics of the performed sport and sex of subjects. These factors also influence the success in using of a neurophysiological feedback while training [48]. In line with our results, it was also shown that in sensorimotor tests, athletes and drummers exhibit a significant difference compared to untrained people and non-drummer musicians [49]. Sport exercises and playing drums cause adaptive effects in sensorimotor function. Short-term training with a brain–computer interface increases the level of desynchronization of the mu-rhythm in imaginary motion [50]. Thus, the development of sEMG-interfaces and specially the algorithms of signal processing should take into account the individual short-term and long-term training abilities of the users and address them at different levels.
